# Potential effects of telbivudine and entecavir on renal function: a systematic review and meta-analysis

**DOI:** 10.1186/s12985-016-0522-6

**Published:** 2016-04-09

**Authors:** Xiaolu Wu, Shaohang Cai, Zhandong Li, Caixia Zheng, Xiulan Xue, Jianyong Zeng, Jie Peng

**Affiliations:** First Affiliated Hospital of Xiamen University, Xiamen, Fujian Province China; Nanfang Hospital, Southern Medical University, Guangzhou, Guangdong Province China; State Key Laboratory of Organ Failure Research, Guangdong Provincial Key Laboratory of Viral Hepatitis Research, Department of Infectious Diseases and Hepatology Unit, Nanfang Hospital, Southern Medical University, No.1838, Guangzhou Avenue North, Guangzhou, 510515 China

**Keywords:** Chronic hepatitis B, Telbivudine, Entecavir, Nucleoside analogs, Renal function, Glomerular filtration rate

## Abstract

**Background:**

To assess the potential effects of telbivudine (LdT) and entecavir (ETV) on renal function in patients with chronic hepatitis B (CHB), we performed a meta-analysis of the relevant data available on these agents to evaluate their effects on the estimated glomerular filtration rate (eGFR) during treatment.

**Methods:**

The PubMed, EMBASE, Scopus, CNKI (China National Knowledge Infrastructure), Cochrane Library, and WanFang databases were searched for relevant articles appearing in the literature up to July 1, 2015. A total of 6 studies (1960 CHB patients) with 1-year eGFR outcomes were retrieved and analyzed.

**Results:**

Generally, the results of the 6 studies analyzed showed that eGFR was improved after LdT treatment, but was decreased after ETV treatment. Using a fixed-effects approach, the change in eGFR was found to be significantly different between LdT and ETV treatment (Z = 3.64; *P* = 0.0003). Whereas the eGFR was slightly decreased with ETV compared with baseline (−1.45 mL/min/1.73 m^2^), the eGFR was improved with LdT (2.99 mL/min/1.73 m^2^) after 1 year of treatment. An overall test of effect in the meta-analysis showed that the eGFR in LdT-treated patients was significantly improved after 1-year of treatment (Z = 3.71; *P* = 0.0002).

**Conclusion:**

This meta-analysis has confirmed that LdT has a renal protective effect whereas ETV does not. However, whether the benefit on renal function outweighs the occurrence of resistance in specific clinical situations is not yet clear.

## Background

Chronic hepatitis B (CHB) is a major public health problem, particularly in middle and low income countries [1]. An estimated 240 million people are chronically infected with hepatitis B virus (HBV) worldwide, and an estimated 650,000 people die annually from end-stage CHB and its complications such as decompensated cirrhosis and hepatocellular carcinoma (HCC). Currently, 5 nucleos(t)ide analogs (NAs) have proved effective for the treatment of CHB (3 nucleoside analogs: lamivudine [LAM], telbivudine [LdT], and entecavir [ETV]; and 2 nucleotide analogs: adefovir dipivoxil [ADV] and tenofovir disoproxil [TDF]). Although their mechanism of action (inhibition of HBV replication) means that NAs cannot eradicate the virus, premature discontinuation of NA treatment may result in severe consequences, including virological relapse and even liver failure. Due to the economic burden of the disease and poor adherence to NA therapy in some Asian countries [2, 3], the Asian Pacific Association for the Study of the Liver (APASL) has suggested that NAs can be stopped when HBeAg seroconversion has been evident for more than 6 months in HBeAg-seropositive CHB patients, and HBV DNA remains undetectable on 3 separate occasions 6 months apart in HBeAg-seronegative CHB patients [1]. However, virological relapse occurs in nearly 50 % of patients after withdrawal of NAs, even when these recommendations are followed [4, 5]. Thus, the European Association for the Study of the Liver (EASL) has suggested that the ideal endpoint of NA treatment is sustained off-therapy HBsAg loss, with or without HBsAg seroconversion [6]. This means that most patients with CHB will require long-term therapy.

In choosing NAs for first-line treatment, factors that need to be considered include safety, the occurrence of resistance, and cost. In terms of safety, nephrotoxicity is an important consideration with this group of drugs [7]. As renal excretion is the primary route of elimination of NAs, they may cause dose-dependent kidney toxicity via various mechanisms, including alterations in renal tubular transporters, apoptosis, and mitochondrial toxicity. In addition, there is also a relationship between HBV and chronic kidney disease (CKD), mainly as a result of immune complex deposition. The European Virgil study has shown that 19 % of patients with CHB have an estimated glomerular filtration rate (eGFR) less than 80 mL/min before initiation of antiviral therapy [8]. Therefore, the renal tolerance of NAs is a particularly important issue during long-term therapy. In this regard, Gane et al. [9] reported a comprehensive analysis of renal function in patients with CHB who received telbivudine (LdT) therapy, the results of which indicated that there was an 8.5 % increase in mean eGFR with LdT. However, the results are different with TDF treatment. In a cohort study of 737 patients with CHB who were treated with TDF, an increase in the serum creatinine concentration of ≥0.3 mg/dL was observed in 3 % patients after a median of 16 months’ therapy [10]. Similarly, in a study of 214 patients with CHB who were treated with either a nucleoside or nucleotide analog for a mean of 2.4 years, the eGFR was found to be decreased in patients with a baseline eGFR <90 mL/min/1.73 m^2^, regardless of the treatment [11].

Thus, the results of studies reporting the renal effects of NAs have been controversial. Although the nephrotoxicity of the nucleotide analogs ADV and TDF has been established [7, 11, 12], reported changes in eGFR with the nucleoside analogs LdT and ETV have been either a decrease or an increase over time [13–17]. However, the limited numbers of patients with CHB included these studies may have biased the results.

The aim of this systematic review was to evaluate the potential renal effects of the nucleoside analogs LdT and ETV in patients with CHB by pooling the available safety and efficacy data on these 2 agents.

## Methods

### Literature search

The PubMed, EMBASE, Scopus, CNKI (China National Knowledge Infrastructure), Cochrane Library, and WanFang databases were searched for relevant articles appearing in the literature up to July 1, 2015. Of these, the CNKI and WanFang databases provide articles in Chinese. The search process was designed to identify trials by using the following terms: ‘hepatitis B’, ‘renal function’, ‘estimated glomerular filtration rate’, ‘nucleoside analogs’, ‘telbivudine’, and ‘entecavir’. Full-text articles and abstracts were obtained for all potentially relevant articles identified, and reference lists in the articles were also searched.

### Study inclusion and exclusion criteria

The following inclusion criteria were used to select studies for review:

1. Randomized, controlled trials, cohort studies, or case-control studies.
2. Study populations comprising patients with CHB, one of the endpoints in whom was renal function.
3. The study interventions were at least one of LdT and ETV.

Exclusion criteria were:

1. The study population was not adults or it included pregnant patients.
2. The patients were co-infected with either hepatitis C or hepatitis D virus or with human immunodeficiency virus (HIV).
3. The study population included patients with serious concurrent medical illnesses, including liver failure, medical evidence of HCC, or a history of organ transplantation.
4. Potentially nephrotoxic drugs other than NAs were used.
5. The study data were not sufficiently complete to fulfil the requirements for meta-analysis.
6. The study interventions did not include either LdT or ETV.
7. The study population had a prior history of antiviral drug therapy, including interferon or NAs.
8. The study disease was not CHB.

### Efficacy endpoints

The efficacy endpoint was the change in eGFR from baseline to 1 year after the start of treatment. Factors associated with renal damage were also evaluated.

### Literature search, review, and data extraction

Z.L. and J.Z. conducted the literature search and reviewed the information retrieved. Another 2 authors (C.Z. and X.X.) independently extracted data from the retrieved studies using a predesigned data extraction form. If necessary, C.Z. and X.X. asked about and confirmed the study data via e-mails to the other investigators. The following data were extracted:

1. The number of patients with CHB.
2. The design of the study.
3. Baseline patient characteristics (age, gender) and the study interventions.
4. Primary and secondary endpoint data after 1 year of treatment (eGFR was calculated by the Modification of Diet in Renal Disease [MDRD] Study equation before and after treatment and other factors associated with renal damage).

### Risk of bias assessment

X.W. conducted the risk of bias assessment based on 7 criteria, including:

1. Random sequence generation (selection bias).
2. Allocation concealment (selection bias).
3. Blinding of participants and personnel (performance bias).
4. Blinding of outcome assessment (detection bias).
5. Incomplete outcome data (attrition bias).
6. Selective reporting (reporting bias).
7. Other bias.

### Study quality assessment

The quality of each study was assessed according to the Quality of Reporting of Meta-Analyses (QUOROM) guidelines. Discrepancies were resolved with the assistance of an arbiter (S.C.) when this was necessary. The quality of the articles was reviewed again at a meeting in which all authors participated, and eligible articles were selected by a consensus of all authors.

### Statistical analysis

Data analysis was performed by using the Review Manager 5.0 (Cochrane Collaboration, Oxford, UK). For each eligible study, dichotomous data were presented as the relative risk (RR) with 95 % confidence intervals (CI), while continuous data were presented as the weighted mean difference (WMD). The meta-analysis was performed using fixed-effect or random-effect methods, depending on the absence or presence of significant heterogeneity [18]. Statistical heterogeneity between trials was evaluated by chi-square (*χ*^2^) and I-square (I^2^) tests, with significance set at *P* < 0.10. In the absence of statistical heterogeneity, the fixed-effect method was used to combine the results. When heterogeneity was confirmed (*P* < 0.10), the random-effect method was used.

Additionally, a sensitivity analysis was performed if low-quality trials were identified. The overall effect was tested using Z scores calculated by Fisher’s Z transformation, with significance set at *P* < 0.05.

## Results

### Literatures search results

**Fig. 1:**
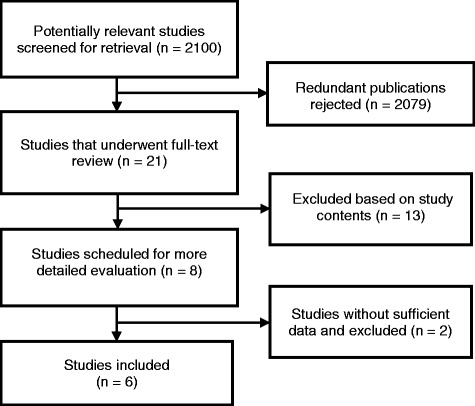
Study retrieval and selection flow chart

A total of 2100 studies were identified by the database searches. By scanning the titles, 1156 redundant publications were excluded and, after referring to the abstracts, a further 923 studies that did not satisfy the inclusion criteria were rejected. The remaining 21 studies underwent full-text review, and 8 were selected for more detailed evaluation (reasons for exclusion of the other 13 papers were: review articles, 3; interventions did not include either LdT or ETV monotherapy, 5; the study population included patients with liver transplantation, 2; the study population included patients with HBV-associated glomerulonephritis, 2; and the treated study population included non-naïve CHB patients, 1). Subsequently, 2 of the 8 selected studies [11, 19] were excluded as the data they provided were insufficient for analysis. Thus, 6 studies [9, 15, 16, 20–22] met the inclusion criteria and were included in the meta-analysis (Fig. 1). The 6 studies involved a total of 1960 patients with CHB (1080 of whom were treated with ETV and 880 with LdT). Two of the studies were RCTs, 3 were cohort studies, and 1 was a case-control study. All 6 studies were published in full-text form.

The characteristics of the selected studies are summarized in Table 1.

**Table 1:** Characteristics of the 6 studies included in the meta-analysis

Study	Region	Study type	Interventions	Sample size	Age^a^ (years)	Gender (F/M)	Follow-up period
Koklu et al. (2015) [16]	Turkey	Case control	LAM/ETV/TDF	LAM: 302	LAM: 49.21 ± 13.17	292/565	2 years
				ETV: 282	ETV: 49.86 ± 13.35		
				TDF: 273	TDF: 47.74 ± 12.45		
Lee et al. (2015) [19]	Korea	Cohort	LdT/ETV	LdT: 116	LdT: 53.6 ± 10.9	229/465	1.5 years
				ETV: 578	ETV: 54.8 ± 11.3		
Jia et al. (2015) [15]	China	Cohort	ADV/ETV	ADV: 165	ADV: 46.2 ± 9.2	95/235	5 years
				ETV: 165	ETV: 48.6 ± 8.7		
Liang et al. (2014) [20]	Taiwan	Cohort	LdT/ETV	LdT: 34	LdT: 46.68 ± 1.87	10/44	1 year
				ETV: 20	ETV: 44.45 ± 1.95		
Yan & Han (2012) [21]	China	RCT	LdT/ETV/ADV	LdT: 50	LdT: 37.3 (22-59)	36/79	1 years
				ETV: 35	ETV: 43.0 (21-64)		
				ADV: 30	ADV: 39.4 (25-62)		
Gane et al. (2013) [9]	Worldwide	RCT	LdT/LAM	LdT: 680	N/A	N/A	2 years
				LAM: 687			

^a^Age expressed as mean ± SD or median (range)

*ADV* adefovir, *ETV* entecavir, *LAM* lamivudine, *LdT* telbivudine, *N/A* not applicable, *RCT* randomized controlled trial, *TDF* tenofovir

### Changes in eGFR with LdT and ETV treatment

**Fig. 2:**
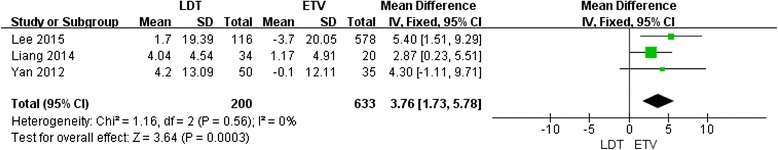
Forest plot of studies included: analysis the change in eGFR after 1 year of treatment in 3 studies directly comparing LdT with ETV

Three studies were designed as comparisons of LdT and ETV [20–22], each of which had no significant differences in baseline demographic data (age, gender) and other patient characteristics (HBeAg status, serum HBV DNA viral load, and eGFR level) between the study groups. A meta-analysis was conducted to compare differences in the eGFR changes after 12 months of treatment with LdT or ETV (Fig. 2). Generally, the results showed that eGFR was improved after LdT treatment (ΔeGFR: 3.31 mL/min/1.73 m^2^) and decreased after ETV treatment (ΔeGFR: −0.88 mL/min/1.73 m^2^). Based on the *χ*^2^ and I^2^ tests in the meta-analysis, a significant heterogeneity was not observed between the treatment groups (*P* = 0.56). Using the fixed-effects approach, changes in eGFR were found to be significantly different in the LdT and ETV treatment groups (Z = 3.64; *P* = 0.0003).

**Fig. 3:**
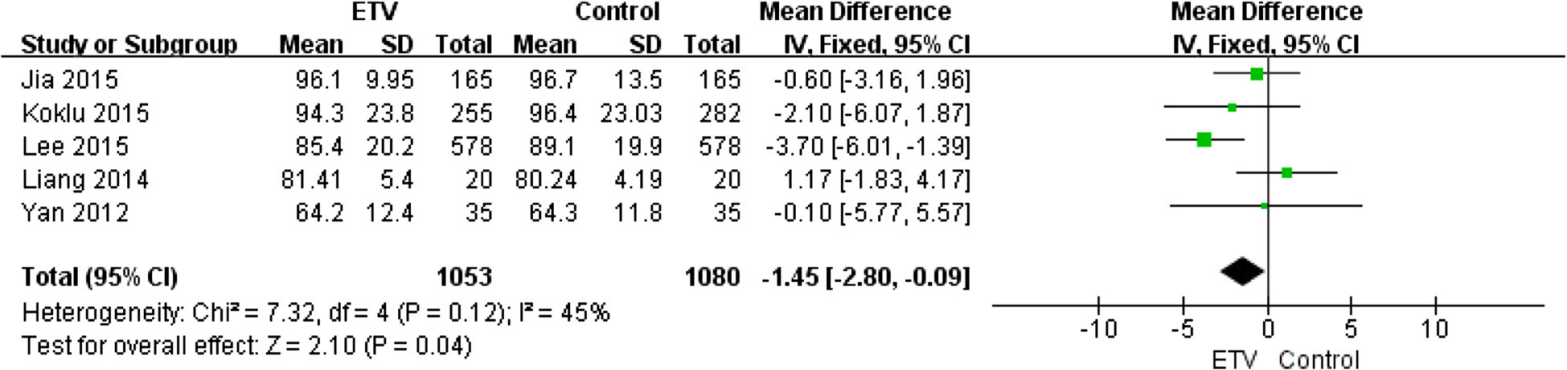
Forest plot of studies included: analysis the change in eGFR after 1 year of treatment in 5 studies of ETV

Five studies [15, 16, 20–22] reported the change in eGFR after 12 months of treatment with ETV (Fig. 3). An unpaired test was used as a substitute for paired tests for these studies due to the lack of before-and-after ETV treatment pairing information in the meta-analysis. The results showed that the eGFR was slightly decreased after ETV treatment compared with baseline (−1.45 mL/min/1.73 m^2^), and the *χ*^2^ and I^2^ tests indicated no heterogeneity (*P* = 0.12). The overall test result based on the standardized mean difference (SMD) and using the fixed-effects approach indicated that the eGFR was significantly decreased after 12 months of treatment with ETV (Z = 2.1; *P* = 0.04).

**Fig. 4:**
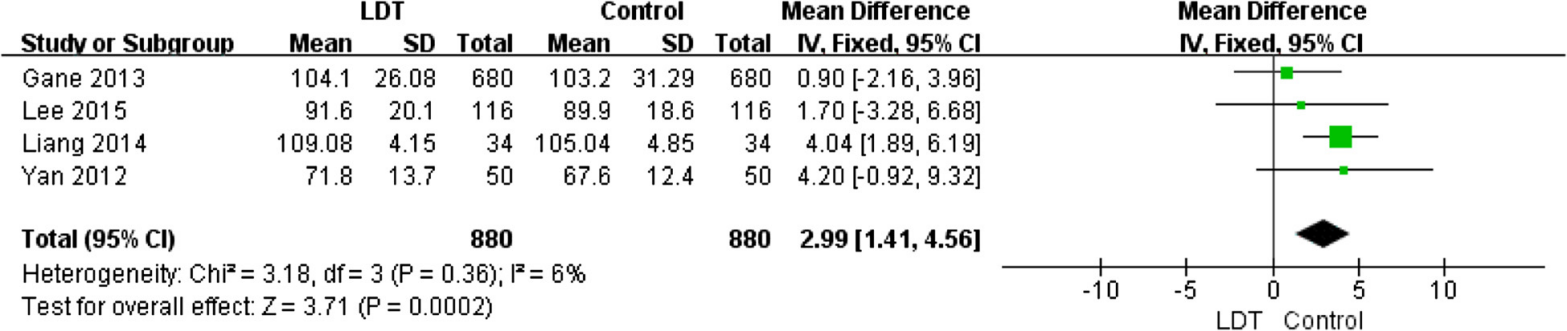
Forest plot of studies included: analysis the change in eGFR after 1 year of treatment in 4 studies of LdT

Four trials [9, 20–22] reported the change in eGFR after 12 months of LdT treatment (Fig. 4). As with the ETV evaluation, an unpaired test was used as a substitute for paired tests for these studies due to the lack of before-and-after LdT treatment pairing information. The results showed that the eGFR was improved by 2.99 mL/min/1.73 m^2^ with LdT after 1 year of treatment. When the fixed-effect model was used, no statistical heterogeneity was observed in the 4 trials (*P* = 0.36). The overall test result indicated a statistically significant change in the eGFR (Z = 3.71; *P* = 0.0002).

**Fig. 5:**
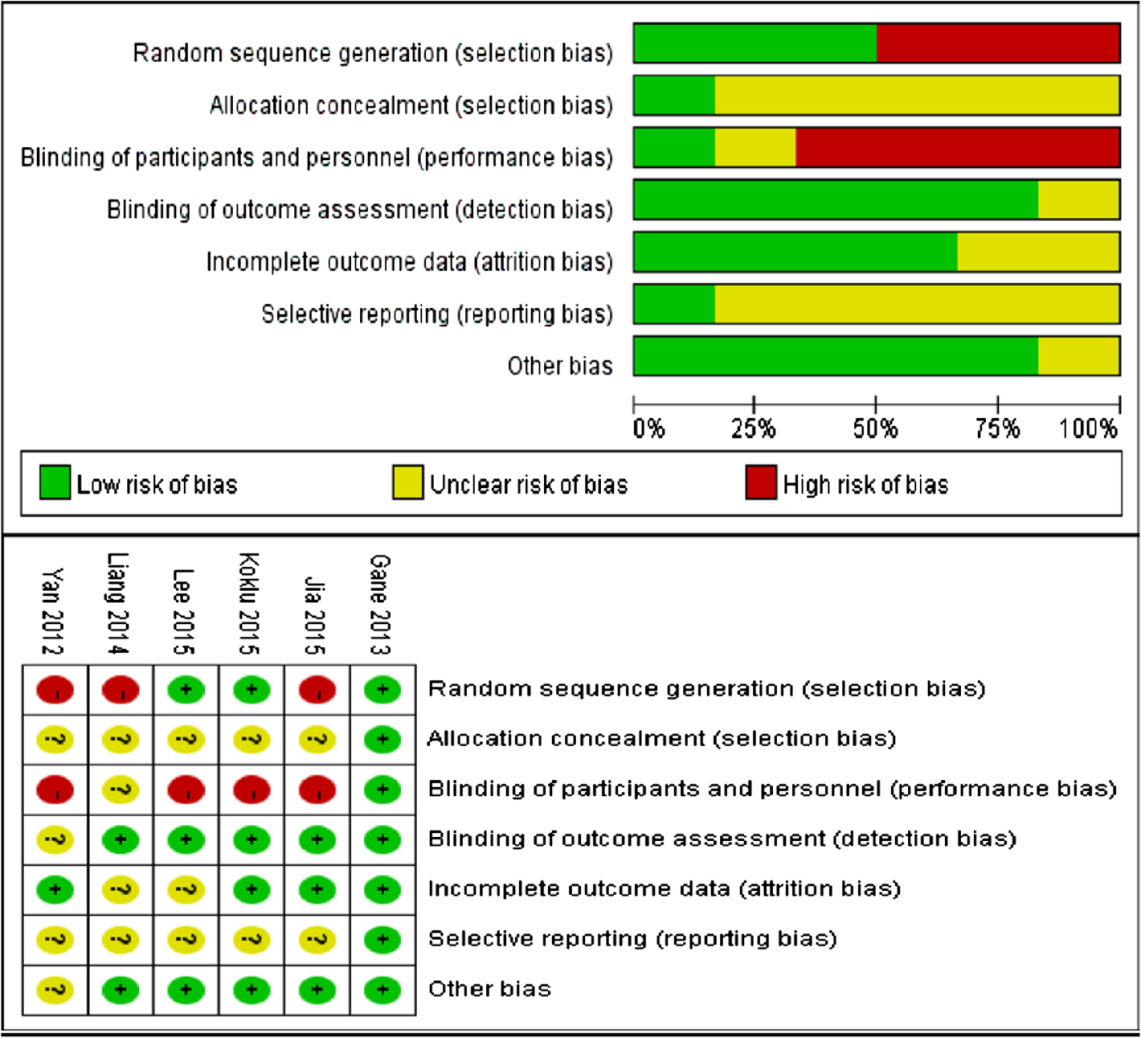
Risk of bias in the 6 studies included in the analysis

The risk of bias assessment conducted for each study included in the meta-analysis is presented in Fig. 5.

### Changes in eGFR in CHB patients with renal function impairment at baseline

CHB patients with hypertension, diabetes, or transplanted livers often exhibit renal function impairment (eGFR <90 mL/min/1.73 m^2^). As there were only limited data on changes in eGFR in patients with impaired renal function at baseline, a meta-analysis was not able to be conducted for this subgroup. However, the study of Lee et al. [20] showed a significantly different outcome in LdT-treated CHB patients with hypertension in comparison with ETV-treated patients. The mean eGFR improved from 84.2 mL/min/1.73 m^2^ at baseline to 86.0, 86.4 and 86.6 mL/min/1.73 m^2^ after 6, 12 and 18 months of treatment, respectively, in the LdT-treated group, while in the ETV-treated group, the mean eGFR decreased from 80.8 mL/min/1.73 m^2^ at baseline to 74.9, 75.7 and 74.8 mL/min/1.73 m^2^ after 6, 12 and 18 months, respectively (*P* = 0.02).

Among CHB patients with diabetes, a similar result was observed: the eGFR increased from 87.0 to 90.0 mL/min/1.73 m^2^ in LdT-treated patients at 18 months, whereas it decreased from 85.5 to 78.5 mL/min/1.73 m^2^ in ETV-treated patients (*P* = 0.001). The mean change in eGFR was a 2.5 % increase after 18 months of LdT treatment, but a reduction of 6 % after 18 months of ETV treatment [20].

A regression analysis by Koklu et al. [16] found that there were no independent factors associated with a shift in the baseline GFR from 60-89 mL/min/1.73 m^2^ to ≥90 mL/min/1.73 m^2^, including the antiviral drug administered (LAM, ETV or TDF), patient age and gender, the presence of hypertension, diabetes and HBeAg, the HBV DNA level, and the fibrosis stage. Another study reported by Gane et al. [9] showed that 72.3 % of patients with baseline chronic kidney disease (CKD) stage 2 (eGFR 60–89 mL/min/1.73 m^2^) who received LdT had an improvement in their eGFR to ≥90 mL/min/1.73 m^2^ after 104 weeks of treatment, as compared with 52.6 % of patients who received LAM. In the latter group, LAM treatment was associated with either a decrease in eGFR or a modest increase [9].

## Discussion

There is a close relationship between CHB and CKD. Data from a real-life cohort study of CHB patients conducted in Germany indicated that 33.3 % had CKD stage 2 prior to starting antiviral treatment [23]. Similarly, a cohort study of 290 Asian patients with CHB found that 35–45 % had CKD stage 2 prior to treatment [24]. HBV-related glomerulopathies are important causes of end-stage renal disease and are a cause for concern.

There are 3 mechanisms by which patients with CHB may develop renal impairment. Firstly, HBV infection may cause renal changes such membranous nephropathy and mesangiocapillary glomerulonephritis via immune complex deposition. Secondly, renal impairment often develops in patients with end-stage CHB disease and liver cirrhosis, the causes of which are multiple and include renal hypoperfusion due to the lack of an effective circulating blood volume. Some studies have reported that renal function impairment is present in 33 % of patients with end-stage liver disease and that this is correlated with a high mortality rate [25–27]. Thirdly, because all currently available NAs are predominantly eliminated in unchanged form by renal clearance, these drugs are considered to be associated with dose-dependent nephrotoxicity, especially the nucleotide analogs. In a study reported by Ha et al. [24], in which patients with CHB were treated with ADV 10 mg daily, ADV was found to be an independent predictor of significant renal dysfunction (eGFR ≤50 mL/min), with a hazard ratio of 3.94. Despite the higher dosage of TDF in comparison with ADV (300 mg daily vs 10 mg daily), nephrotoxicity is observed less commonly with TDF than with ADV. A study of 114 patients with baseline eGFR <90 mL/min who were grouped into 3 CKD stages (CKD stage 2 [eGFR 60–89 mL/min]; CKD stage 3 [eGFR 30–59 mL/min]; and CKD stage 4 [eGFR 15–29 mL/min]) showed that treatment with TDF did not significantly alter renal function after 48 weeks of treatment (mean eGFRs: 76 vs 77 mL/min, 50 vs 49 mL/min, and 23 vs 23 mL/min in patients with CKD stages 2, 3 and 4, respectively) [28]. However, despite the lack of significant eGFR changes, a potential effect of TDF on proximal tubules still needs to be considered, as signs of proximal tubular damage in CHB patients receiving TDF treatment are not rare [7]. A severe adverse effect that has often been reported with TDF is Fanconi syndrome [29]. It is also believed that ADV may be directly toxic to proximal tubular cells. Hyperphosphaturia has been shown to occur in approximately one-third of untreated patients with CHB, and this was worsened in approximately 25 % of patients with TDF treatment without causing significant hypophosphatemia [30].

The potential renal impact of nucleoside analogues such as ETV and LdT has also emerged as a concern recently. Although several studies have shown beneficial renal effects with LdT monotherapy [9, 20–22], the effect of ETV on renal function is more controversial, as data from several studies have shown that eGFR is either increased or stable or slightly decreased after ETV treatment [15, 16, 20–22]. This is the first meta-analysis to evaluate the impacts of ETV and LdT on renal function in patients with CHB. Its results indicate that LdT treatment improved the eGFR of CHB patients by an average 2.99 mL/min/1.73 m^2^, while ETV treatment decreased the eGFR by an average 1.45 mL/min/1.73 m^2^. This result confirms that LdT has a renal function protective effect, whereas ETV does not. The mechanism responsible for the improvement of renal function during LdT treatment is still under investigation. Currently available evidence indicates that LdT can be considered a suitable NA for the treatment of CHB patents with a high risk of renal impairment. In contrast, our results indicate that ETV treatment of CHB patients slightly decreases renal function after 1 year of treatment. However, this does not constitute direct evidence to prove that ETV causes renal toxicity, since blank control group data are missing. The normal decline in renal function due to aging needs to be taken into consideration as a reason for the change of eGFR with ETV treatment. Nevertheless, the results of our study do suggest that CHB patients with a high risk of renal impairment should have their eGFR monitored during long-term treatment with ETV, and medical rescue measures such as dosage adjustment should be instituted when necessary.

The Asian-Pacific consensus statement on the management of CHB indicates that the type of drug therapy in patients with renal dysfunction can be similar to that in patients without renal dysfunction [1]. Since all of the currently available NAs are eliminated unchanged in the urine, their dosages should be adjusted in patients with a GFR <50 mL/min. Half the usual daily dose or a full dose on alternate days should be given if the GFR is 30–49 mL/min; a dose every 3 days should be given if the GFR is 10–29 mL/min; and after dialysis, a dose should be given weekly. Data from our meta-analysis show that ETV monotherapy causes a significant but slightly decreased eGFR in patients with CHB. This finding indicates that patients receiving long-term ETV treatment have a potential need for dosage adjustment. Thus, renal function needs to be monitored frequently during long-term ETV treatment, and the requirement for dosage adjustment constantly considered, especially in older patients and those with renal impairment or suffering from other diseases such as hypertension and diabetes. In a retrospective, single-center, matched case-control study of 115 CHB patients, Tsai et al. reported that both LdT-treated and ETV-treated patients with baseline eGFR values <90 mL/min/1.73 m^2^ had improvements in their eGFRs after 1 year of treatment [31]. Qi et al. [14] reported that 3 patients with mild renal impairment at baseline had normal renal function after LdT treatment, and suggested that LdT may reverse renal dysfunction; in contrast, 2 ADV-treated patients with mild renal impairment at baseline progressed to moderate renal impairment [14]. The study of Cholongitas et al. [32], which included 42 adult patients with liver transplants for HBV-related cirrhosis who were treated with a combination of NAs and hepatitis B immune globulin (HBIG), showed that LdT treatment was associated with an improvement in GFR in comparison with non-LdT treatment (mean changes in GFR, +2.5 mL/min vs −4.2 mL/min, respectively). The authors concluded that even in liver transplant recipients with a relatively low GFR who are receiving nephrotoxic drugs concomitantly and in whom risk factors for renal impairment co-exist, LdT may be associated with significant improvement in renal function [32].

Factors predicting significant changes in eGFR during NA treatment have been reported in some studies. Qi et al. [14] showed that independent predictors of a ≥20 % increase in eGFR were age, eGFR at baseline, and LdT treatment, while predictors of a ≥20 % decrease in eGFR were age, sex, body mass index (BMI), eGFR at baseline, and ADV treatment [14]. Similar results were observed in the study by Lee et al. [33] which included 1043 patients with CHB treated with ADV, LdT, LAM, or ETV or a combination of these drugs. In this study, age, gender, baseline eGFR, and the type of treatment were significant predictors of eGFR changes over time, while the baseline eGFR was the variable most capable of predicting an eGFR decrease [14]. Another study reported by Qi et al. [17], which included 195 patients with CHB treated with combination therapy with either LAM + ADV, LdT + ADV, or ETV + ADV, showed that age (OR, 1.03; *P* = 0.047), sex (OR, 0.26; *P* = 0.009), eGFR at baseline (OR, 0.92; *P* < 0.001), and treatment with LAM + ADV (OR, 6.30; *P* = 0.001) were all independent predictors of a >20 % decrease in eGFR. In the group treated with LdT + ADV, a decrease in eGFR was not observed but rather was slightly increased over 3 years of continuous treatment [17]. Intriguingly, a multivariate analysis in the study reported by Jia et al. [15] indicated that old age (*P* = 0.003) and ADV treatment (*P* = 0.005) were significant predictors of a urinary β2-M abnormality.

As well as decreased glomerular function, proximal renal tubular injury associated with nucleotide analogs has also received attention recently. Fanconi syndrome with hypophosphatemia, hypouricemia, aminoaciduria, and glycosuria has been reported with ADV and TDF [34]. Qi et al. [17] found that CHB patients treated with LdT + ADV had a slightly increased eGFR over 3 years of continuous treatment. These findings suggest that LdT may be an appropriate choice as rescue therapy when patients experience renal impairment during long-term ADV or TDF treatment. However, whether LdT can improve proximal renal tubular function, and whether LdT + TDF combination therapy can exert a good clinical effect on both virology and nephrology outcomes are unanswered questions.

HBV-associated membranous nephropathy, an extrahepatic HBV infection, is one of the most common causes of secondary membranous nephropathy [35, 36]. In most cases, the manifestations of HBV-associated membranous nephropathy are the nephrotic syndrome and mild-to-moderate proteinuria with hematuria [37]. Although a beneficial effect of LAM treatment has been established, this is offset by a low barrier to the emergence of drug resistance. LdT has been shown to cause more potent suppression of HBV than LAM [38], but the available data on LdT monotherapy and LdT + TDF combination therapy in HBV-associated membranous nephropathy are still limited. Thus, despite its protective effect on renal function, whether LdT can be regarded as an appropriate treatment option for HBV-associated membranous nephropathy requires further investigation. As the barrier to drug resistance with LdT is not high, the 2-year risk of resistance with LdT has been reported to be 25.1 % in HBeAg-seropositive patients with CHB, and 10.8 % in HBeAg-seronegative patients [39]. Therefore, there is still a concern about switching to LdT treatment in patients who have been receiving long-term TDF treatment and have serum HBV DNA levels below detectable limits. In this regard, drug resistance occurs more frequently in CHB patients who exhibit bad adherence to NA treatment and have a high viral load

Whether the improvement of eGFR with LdT outweighs the risk of drug resistance, myopathy, and peripheral neuropathy is an unanswered question. Since LdT has a low barrier to drug resistance, and is similar in this respect to LAM, LdT should not be used as a first-line drug in CHB patients with normal renal function. However, in CHB patients with renal impairment and a low risk of developing drug resistance, LdT can be considered.

A limitation of this analysis was the low number of studies providing relevant data, as the extrahepatic effects of NAs are often ignored. Another limitation is the bias within and across the studies included, which may also affect the study results. Since there only 6 studies were included in the meta-analysis, the heterogeneity statistic I^2^ may be biased [40, 41]. Also, eGFR was evaluated after only 1 year. As the duration of treatment of patients with CHB is several years, the clinical significance of the reported eGFR changes needs further elucidation.

## Conclusions

In conclusion, this meta-analysis provides evidence that LdT has a renal protective effect whereas ETV does not. However, the mechanism of the renal protective effect of LdT is not clear, and nor is it clear whether the benefits of LdT on renal function outweigh its lower barrier to resistance in specific clinical situations. Additionally, when and how the dosage of ETV should be modified during long-term treatment in patients with renal impairment, especially those with co-existing hypertension and diabetes, are other unanswered questions. These questions need to be addressed in well-designed clinical trials to explore and thus potentially modify the existing guideline recommendations.

## Abbreviations

ADV: adefovir dipivoxil
ALT: alanine aminotransferase
CHB: chronic hepatitis B
CKD: chronic kidney diseases
CNKI: China National Knowledge Infrastructure
eGFR: estimated glomerular filtration rate
ETV: entecavir
HBeAb: hepatitis B e antibody
HBeAg: hepatitis B e antigen
HBsAb: hepatitis B surface antibody
HBsAg: hepatitis B surface antigen
HBV: hepatitis B virus
LAM: lamivudine
LDT: telbivudine
NAs: nucleos(t)ide analogs
TDF: tenofovir
